# Wanted: more monitoring and control during inclusion body processing

**DOI:** 10.1007/s11274-018-2541-5

**Published:** 2018-10-19

**Authors:** Diana Humer, Oliver Spadiut

**Affiliations:** Research Area Biochemical Engineering, Institute of Chemical, Environmental and Bioscience Engineering, TU Wien, Gumpendorfer Straße 1a, 1060 Vienna, Austria

**Keywords:** *E. coli*, Fed-batch, Inclusion bodies, Monitoring, Process analytical technology

## Abstract

Inclusion bodies (IBs) are insoluble aggregates of misfolded protein in *Escherichia coli*. Against the outdated belief that the production of IBs should be avoided during recombinant protein production, quite a number of recombinant products are currently produced as IBs, which are then processed to give correctly folded and soluble product. However, this processing is quite cumbersome comprising IB wash, IB solubilization and refolding. To date, IB processing often happens rather uncontrolled and relies on empiricism rather than sound process understanding. In this mini review we describe current efforts to introduce more monitoring and control in IB processes, focusing on the refolding step, and thus generate process understanding and knowledge.

## What are inclusion bodies?

*Escherichia coli* is a widely used production host for recombinant proteins. However, due to its reducing environment in the cytoplasm and the metabolic burden posed by recombinant protein production, most products of interest (POI) aggregate to incorrectly folded particles, termed inclusion bodies (IB). They occur in the polar region of the cell and are characterized by a porous structure, hydration, spherical or rod-shaped appearance and a diameter of about 1 µm (Bowden et al. 1991; Rinas et al. 2017). In the past, many protocols aimed at avoiding IB formation during heterologous protein production. These so-called midstream approaches, like chaperone co-expression, reduced growth temperature, expression tuning, strains that promote soluble POI production, cofactor addition to the medium, fusion proteins and translocation to the periplasm, are extensively discussed elsewhere (Basu et al. 2011; Burgess 2009; García-Fruitós et al. 2012; Kaur et al. 2017; Sørensen and Mortensen 2005). Albeit, these attempts rarely resulted in sufficient yields of soluble POI to be relevant for industrial purposes.

However, the vision of IBs changed in the past few years: recent studies reported that enzyme activity is maintained to 11–100% in IBs depending on the protein in question (Gatti-Lafranconi et al. 2011). Thus, versatile, direct applications for IBs have been developed ever since: immobilized catalysts, scaffolds in tissue engineering, models for amyloidosis and prion propagation, functional materials in tissue engineering, targeted and non-targeted drug delivery systems or implantable depots of therapeutic proteins (Rinas et al. 2017).

**Fig. 1:**
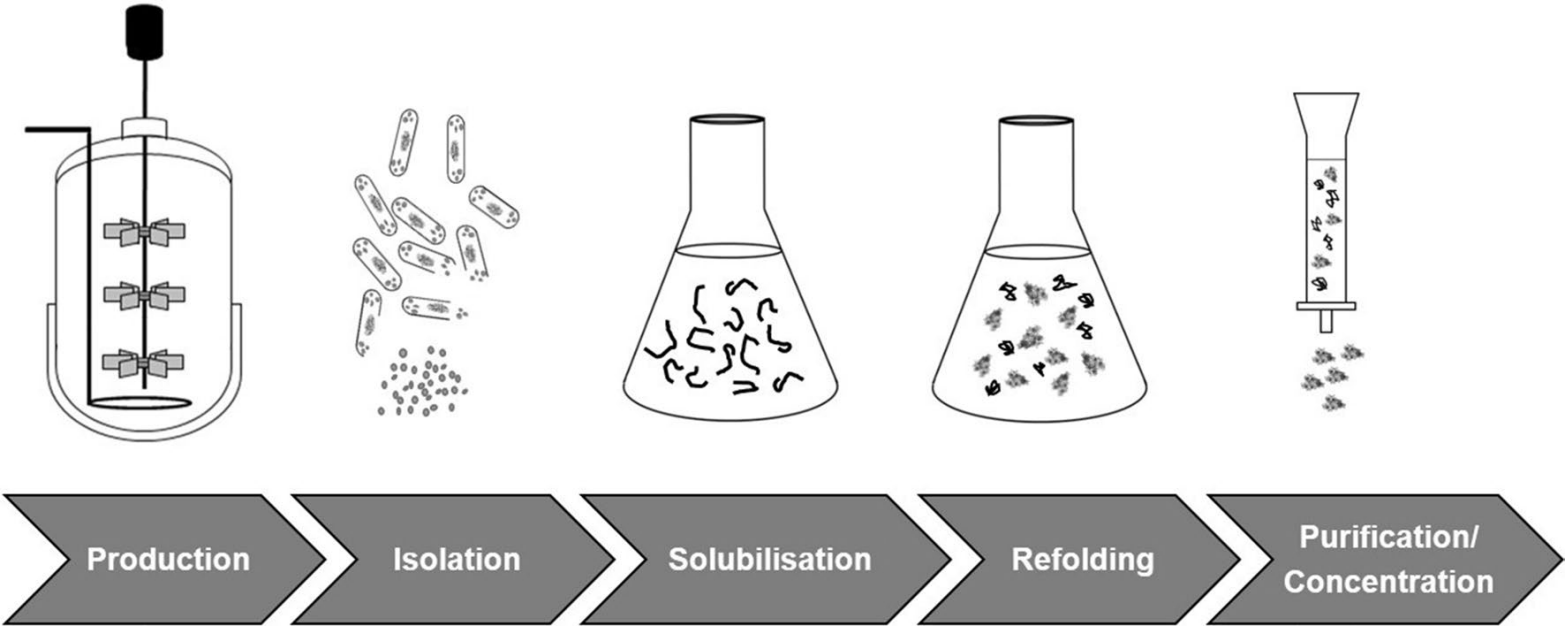
Schematic inclusion body process

Furthermore, the fast emerging market of biosimilars in the biopharmaceutical industry has a high demand for recombinant proteins. Therefore, it is of utmost importance to enhance the yield and lower the production expenses for POIs to enable economic industrial production. Considering this, IB production is an option with several advantages, like high product yield, up to 95% POI purity within the IBs, high mechanical and thermal stability and resistance to proteases (Eggenreich et al. 2016; Rinas et al. 2017). Currently, a growing number of recombinant products is produced as IBs, which are then processed into soluble product (Table 1). A state-of-the-art IB process is schematically depicted in Fig. 1. At first, the IBs are produced by *E. coli* fermentation, the cells are harvested by centrifugation and are lysed. Subsequently, consecutive IB washing steps are performed, before the IBs are solubilized with denaturants, like urea, sarkosyl or guanidine hydrochloride, and the cell debris is removed by centrifugation. After solubilization, the POI needs to be refolded in its native conformation which is usually achieved by dilution, dialysis or on-column refolding with the aid of small molecule additives (Alibolandi and Mirzahoseini 2011; Ling et al. 2015). Finally, the refolded POI might undergo a purification step to further increase purity. A more detailed IB process description can be found elsewhere (Basu et al. 2011; Burgess 2009; Eggenreich et al. 2016; Hoffmann et al. 2017; Kaur et al. 2017; Rathore et al. 2013; Singh et al. 2015).

**Table 1:** Prominent examples of recombinant products that are produced as inclusion bodies

Protein	Applications	References
β-Galactosidase	Molecular biology, milk industry	Worrall and Goss (1989)
GFP	Molecular biology	Vera et al. (2007)
Endoglucanase	Biotechnology industry	Tokatlidis et al. (1991)
d-Amino acid oxidase	Potential target molecule for the treatment of chronic pain and schizophrenia	Nahalka and Nidetzky (2007)
Lipase	Digestion enzyme for enzyme therapy	Ami et al. (2005)
Polyphosphate kinase	Sialylation of biotherapeutic glycoproteins	Nahálka and Pätoprstý (2009)
Maltodextrin phosphorylase	Starch degradation	Nahálka (2008)
Sialic acid aldolase	Influenza antivirotics	Nahálka et al. (2008)
Insulin	Diabetes therapy	Nilsson et al. (1996) Williams et al. (1982)
Lysozyme	Food industry, molecular biology	Batas et al. (1999)
Recombinant immunotoxins	Anticancer drugs	Linke et al. (2014)
Maize transglutaminase	Clinical, food additives, wool textiles, biopolymers	Carvajal et al. (2011)
Human granulocyte colony stimulating factor (GCSF)	Chemotherapy induced neutropenia	Kateja et al. (2017) Pathak et al. (2016)
Mink (mGH) and porcine (pGH) growth hormones	Food industry, fur industry	Bajorunaite et al. (2009)
Papain	I.a. clarifying beer, detergents, meat tenderization, blood coagulant, gastritis, removal of necrotic tissue, tetanus vaccines	Choudhury et al. (2009)
Papain-like cysteine proteases	May represent viable drug targets for major diseases	Ling et al. (2015)
Human immunodeficiency virus type-I protease	Potential target for the development of antiviral agents for HIV	Cheng et al. (1990)
Major capsid protein of human papillomavirus type 16 (HPV 16)	HPV 16 vaccine	Choe et al. (2003)
Interleukin-13 (IL-13)	NMR studies of function	Eisenmesser et al. (2000)
Recombinant major wasp allergen Antigen 5 (Ves v 5)	Diagnostic and therapeutic applications for type 1 allergic diseases	Kischnick et al. (2006)
Human alpha-fetoprotein (rhAFP)	Immunomodulation, treatment of several autoimmune diseases	Leong and Middelberg (2007)
Recombinant human vascular endothelial growth factor (rhVEGF)	Cell culture	Pizarro et al. (2009)

## Challenges in IB processing

Although the benefits of IB-based recombinant protein production are manifold, some challenges are yet to be mastered. The state-of-the-art procedure for IB processing still relies heavily on empirical approaches instead of Quality-by-Design (QbD). A major problem are non-competitive yields because of low recovery rates. Commercial IB processes often employ an uncontrolled dilution method with immense buffer volumes requiring huge refolding vessels (Rathore et al. 2013). It is known that mixing times increase with scale because uniform stirring is challenging in big tanks. Hence, fluctuations in concentrations in the refolding buffer often cause agglomerate formation and fragmentation (Pan et al. 2014; Pizarro et al. 2009). Thus, the space-time-yields (STY) for IB processes are very low.

Since the most crucial step in IB processing is the refolding step, we will focus on methods for the precise monitoring during this unit operation in this mini review. We address the question: “Which tools do we need to convert trial-and-error to sound knowledge brought about by intelligent in-process monitoring during protein refolding?”.

## Monitoring tools

In general, biopharmaceutical processes have to be reproducible, robust, scalable, cheap and safe, while still generating high product yields. To meet these challenging requirements, constant process monitoring allowing in-process control has to be implemented. In this respect the so-called process analytical technology (PAT) was launched by the US Food and Drug Administration (FDA) to enable better process understanding and to facilitate compliance with regulatory requirements (FDA 2004). The overall goal of PAT is to accomplish process knowledge by real-time process monitoring (Read et al. 2010a, b). Currently, different methods are available to monitor the IB refolding process and analyze its efficiency (Table 2).

**Table 2:** Methods to monitor inclusion body refolding

Method	Information	Necessary protein amount	Advantages	Limitations	References
Circular dichroism (CD)	Far UV-CD: secondary structure composition Near UV-CD: tertiary structure	Far UV-CD: 0.25 g/l Near UV-CD: 2.5 g/l	Measurement can be performed in physiological buffers Non-destructive Measurement time 30 min or less	No residue-specific information Not compatible with high amounts of denaturants Non-detergent sulfobetaine (NDSB) cannot be used	Kelly et al. (2005) Greenfield (2006)
Dynamic light scattering (DLS)	Tertiary and quaternary structure Quantification	0.05 g/l	Rapid No calibration or buffer blanking Can be used for all proteins	Low resolution Samples need to be free of other particles	Bhattacharjee (2016) Yu et al. (2013)
Dissolved oxygen and redox probes	Soft sensor to monitor refolding of proteins with disulfide bridges	Independent of protein concentration	Rapid Simple Cost-efficient DO sensor can be correlated to product quality	Technical issues like signal to noise variability or probe fouling	Pizarro et al. (2009)
Electrospray ionisation–ion mobility spectrometry–mass spectrometry (ESI–IMS–MS)	Monitoring of oxidation process and study aggregation	Ca. 1.5 g/l for 50 kDa protein	Analyze and quantify a mixture of proteoforms during folding (e.g. different disulfide bonds)	Expensive equipment Involatile sample buffers are not compatible with ESI–IMS–MS	Furuki et al. (2017) Young et al. (2016)
Extrinsic fluorescence	Tertiary and quaternary structure	Ca. 0.015 g/l for 50 kDa protein	Sensitive Suitable for high-throughput screening	Dye might interfere with protein aggregation	Hawe et al. (2008) Younan and Viles (2015)
Fourier transform infrared spectroscopy (ATR-FTIR)	Secondary structure and dynamics	> 0.01 g/l	Tolerant to salt and sample turbidity Can be used for all proteins High wavelength precision	No time-related data	Pathak et al. (2016) Walther et al. (2014) Baldassarre and Barth (2014)
Nuclear magnetic resonance spectroscopy (NMR)	Tertiary and quaternary structure	> 25 g/l for 50 kDa protein	Real-time application possible Non-destructive Structure can be analyzed under native conditions	Large number of samples necessary Limited to small proteins (≤ 40 kDa) or protein fragments	Lanucara et al. (2014) Kelly et al. (2005)
Reversed phase high performance liquid chromatography (RP-HPLC)	Monitor unfolded proteins and primary structure	> 0.3 g/l	Rapid High resolution Robust	High temperature during analysis may lead to aggregate formation Mere chemical information because proteins are denatured	Sturaro et al. (2016) Herman et al. (2002)
Size exclusion high performance liquid chromatography (SE-HPLC)	Quantitative protein analysis	0.012 g/l	Non-destructive Sensitive Non-denaturating elution conditions possible	Limited dynamic range Inaccuracy due to alteration of size distribution	Codevilla et al. (2004) Brusotti et al. (2017)
Raman spectroscopy	Secondary structure Protein quantification (SERS)	> 1 g/l > 0.08 g/l for SERS	Sensitive and structural selective	Expensive equipment (CW-UV laser) and complicated instrumentation	Balakrishnan et al. (2008) Eryilmaz et al. (2017) Wen (2007)
Zeta potential analysis	Initial stage of refolding and primary structure	For particle size below 10 nm: > 0.5 g/l For particle size 10–100 nm: > 0.1 g/l For particle size 100 nm–1 µm: > 0.01 g/l	Simple Fast	Very sensitive to dust Cannot detect charge changes during secondary structure formation	Pathak et al. (2016) Panalytical (2013)

*Far UV-circular dichroism* is used to investigate the secondary structure of proteins (measures spectra of β-sheets and α-helices; 190–250 nm) and is recommended for globular proteins rich in α-helices (Pathak et al. 2016). It clearly separates refolded from denatured proteins (Leong and Middelberg 2007). Disadvantages are the need of rather high protein concentrations and limitations in refolding buffer composition (Ling et al. 2015). Furthermore, FAR UV-CD is also limited by the intrinsic insolubility of the samples, which is responsible for a high level of light scattering disturbances and signal loss (Gatti-Lafranconi et al. 2011). Near UV-CD on the other hand can be applied to monitor the tertiary structure (250–300 nm) as it measures aromatic amino acid residues and disulfide bonds (Leong and Middelberg 2007). The amount of protein necessary for UV-CD is about 0.1 mg (Far UV) and 1 mg (Near UV) which amounts to 0.25 g/l and 2.5 g/l, respectively (considering a sample volume of 400 µl).

*Nuclear magnetic resonance spectroscopy* (*NMR*) is a method that gives information about the time needed until the native state of a protein is established (Pathak et al. 2016). It can be used in real-time and monitors the tertiary and quaternary structure of a protein. A drawback of NMR is the fact that it is limited to small proteins below 40 kDa. Besides, protein concentrations should be 0.5 mM or higher (Kelly et al. 2005) and samples have to be purified and concentrated before measurement (Lanucara et al. 2014).

*Reversed phase high performance liquid chromatography* (*RP-HPLC*) can distinguish minor differences in hydrophobicity, therefore it is able to measure levels of product-related impurities, like oxidized and reduced species, in the refolding mix. Oxidized impurities mostly consist of proteins that have formed non-native disulfide bridges. As RP-HPLC is usually carried out at destabilizing conditions (e.g. high temperature) it cannot be used to investigate secondary or tertiary protein structures, which is why the information is of pure chemical nature (Pathak et al. 2016). Moreover, elevated temperature can lead to aggregate formation. Nevertheless, RP-HPLC is a frequently used technique because it is fast, robust and has high resolution (Herman et al. 2002). The amount of protein for RP-HPLC should be more than 0.3 g/l assuming a sample volume of 2 µl (Sturaro et al. 2016).

Another method to investigate aggregation and fragmentation during the refolding process is *size exclusion HPLC* (*SE-HPLC*). Different product species, like dimers, oligomers and fragments, can be distinguished. Especially the combination of UV and fluorescence detection provides detailed information on protein folding, size analysis and quantification (Printz and Friess 2012). Cowan et al. (2008) showed that analytical SE-HPLC is able to quantify the recovery of a monomeric protein down to 0.05 g/l, whereas Codevilla et al. (2004) reached a detection limit of 0.012 g/l for granulocyte colony-stimulating factor.

*Raman spectroscopy* provides information about disulfide bonds and solvent accessibility of specific amino acid side chains (Gatti-Lafranconi et al. 2011). This method is sensitive and structural selective, “it can provide unique insights into protein dynamics” and is able to investigate slow changes in protein conformation (Balakrishnan et al. 2008). Traditional Raman spectroscopy needs a sample concentration of more than 1 g/l (Wen 2007), whereas surface enhanced Raman spectroscopy (SERS) is more sensitive (> 0.08 g/l) (Eryilmaz et al. 2017).

*Zeta potential analysis* is used to determine the surface charge of proteins. It is “a measure of the magnitude of electrostatic repulsion and/or attraction between two molecules and is known to affect protein stability” (Pathak et al. 2016). This method is fast and simple but on the other hand very sensitive to dust. For particle sizes ranging from 100 nm to 1 µm the minimal protein concentration is 0.01 g/l (Panalytical 2013). However, Pathak et al. (2016) reported that zeta potential analysis was not sensitive enough to measure changes in charges during formation of secondary and native structures.

The sensitivity and versatility of *extrinsic fluorescence* makes it suitable for high throughput screening (Pathak et al. 2016; Printz and Friess 2012). Dyes are covalently attached to the POI and variations in surface hydrophobicity can be detected with increased fluorescence intensity during refolding. It is possible to characterize refolding and detect aggregation. A drawback is the possibility of interference with protein aggregation caused by the dye itself (Hawe et al. 2008). Extrinsic fluorescence is very sensitive, a detection limit of 0.3 µM was reported by Younan and Viles (2015).

*Electrospray ionization–ion mobility spectrometry–mass spectrometry* (*ESI–IMS–MS*) is a rapid, robust and sensitive method for conformational analysis of proteins with regard to disulfide bond formation. The technique can quantify a mixture of proteoforms, e.g. different disulfide bond formation during refolding. ESI–IMS–MS can function as a real-time application to investigate protein folding and may be very useful as a PAT tool (Furuki et al. 2017). A disadvantage of ESI–IMS–MS is that involatile buffers (e.g. Tris/HCl) are not compatible with the method and a buffer exchange has to be performed. Protein concentrations as low as 32 µM can be measured by ESI–IMS–MS (Young et al. 2016).

Walther et al. (2014) investigated the usability of *attenuated total reflectance Fourier transform infrared spectroscopy* (*ATR-FTIR*) for inline monitoring of protein refolding. FTIR is tolerant to salt solutions and turbidity of samples. Moreover, the wavelength precision of FTIR is a great advantage because it allows the substraction of water, which is a strong infrared absorber. According to Walther et al. (2014) these properties make it suitable for monitoring unfolding and refolding of native secondary structures. They used an in-situ ATR-FTIR sensor that provided structural but not time-related data of the refolding process. “Inline ATR FTIR enables earlier and more controlled termination of refolding processes, as it is a good method to monitor unwanted aggregation”. Especially the great sensitivity of FTIR to detect individual differences in secondary structure elements makes it highly suitable for inline PAT applications. ATR-FTIR can be applied to any protein since there are no limitations caused by specific protein characteristics. Normally, ATR-FTIR measurements need a protein concentration of 0.01 g/l or higher, but this can be reduced tenfold by isolating the amide I region with filters (Baldassarre and Barth 2014).

Yu et al. (2013) used *dynamic light scattering* (*DLS*) to monitor the refolding process of a protein-based vaccine candidate. As this method has no need for an intrinsic or extrinsic fluorophore, it is not restricted to proteins with distinct properties. Moreover, DLS is rapid (results available within a minute) and has no need for calibration or buffer blanking which makes it a promising online method. Further advantages are that aggregation can be studied with a great variety of solvents including denaturating chemicals and no sample dilution or conditioning is necessary. As DLS measurements are non-intrusive, aggregation or dissociation caused by the assay is greatly decreased. Concerning the precision of the rH values (particle size information) Yu et al. (2013) reported that a difference of 1.5 nm is reliable. Protein concentrations of 0.05–0.1 g/l were reported to give the best results (Bhattacharjee 2016). Opposed to circular dichroism, DLS does not rely on a correlation between tertiary and secondary structures. This is important for oligomeric proteins, because the secondary structure of monomer and oligomer can be hard to distinguish. A small drawback is that the samples need to be filtered because particles and lipopolysaccharide micelles have to be removed before the measurements. Yu et al. (2013) found that DLS data can also be used for quantification of refolded protein because the obtained measurements can be correlated to SE-HPLC results. In summary, DLS has many characteristics making it an ideal PAT tool.

Aeration is a rate-limiting factor for the refolding of recombinant human vascular endothelial growth factor (rhVEGF). Therefore, Pizarro et al. (2009) used inline sensors to observe the percentage of *dissolved oxygen* (*DO*) and the *oxidation reaction potential* (*ORP*) during refolding. Process samples were further analyzed by RP-HPLC. The usage of these probes as online sensors is advantageous because of their simplicity and cost-efficiency. The combined usage of DO and ORP is beneficial because of reduced technical challenges when two probes are used at once (e.g. signal to noise variability, drifting or unexpected failure). Refolding of rhVEGF depends on redox chemistry because this protein is a homodimer with 16 disulfide bonds and Pizarro et al. (2009) showed that there is a direct correlation between the DO sensor and product quality. Therefore, DO online sensors may be used as a platform for proteins with disulfide bridges.

## Recommendations and outlook

In general, the implementation of real-time online sensors (in situ) or near real-time inline analyzers which can be in situ (sampling bypass) or ex situ (application of a sampling module, sample is discarded afterwards) (Whitford and Julien 2007) is favored, since data are available quickly allowing instant process parameter adjustments. Online sensors need to fulfill certain requirements such as the need to provide sufficient sensitivity, linearity and resolution. Furthermore, they should be inert, sterilisable, cleanable, robust, easy to calibrate, free of interference and cost-efficient (Whitford and Julien 2007). It is also of significance to tackle current problems, like signal to noise variability, drifting, probe fouling or unexpected failure of the sensor, to pave the way for universal usage. In part, these problems have already been addressed: probe fouling (protein aggregation on the surface) can be reduced by stirring, addition of chaotropes and attaching diamond crystals near the housing of the probe (Walther et al. 2014). Other issues, like drifting, can be diminished by using two probes simultaneously as mentioned above. Hence, it would be most favorable to use simple soft sensors, like DO and ORP, which allow online monitoring of the refolding process.

In general, monitoring includes the collection of information by measurements with subsequent data processing. For this processing step model-based methods should be applied as they facilitate process understanding and thus the implementation of QbD. Modelling is a tool for the detection and characterization of the relationship between critical process parameters (CPP), key process parameters (kPP) and the generation of process knowledge (Kroll et al. 2017). CPPs define product quality, whereas kPPs also influence the productivity and economical viability (Rathore and Winkle 2009).

The methods presented in this mini review are applicable for in-process monitoring of protein refolding. A mass balance is generated which is able to describe the refolding process at all time points. Then experiments are performed and the data are used to adjust and optimize the model according to the measured values. Once established, model-based methods are valuable for real-time process monitoring because they allow the adaption of process parameters based on predictions, which ensures consistent manufacturing conditions.

Currently, the majority of investigations concerning protein refolding are performed in batch-mode. However, a fed-batch approach was shown to increase the refolding yield (Linke et al. 2014; Mannall et al. 2007). The use of a controlled refolding vessel and application of fed-batch refolding can diminish many current problems: the reduction of buffer volumes, decreased misfolded and aggregate species and enhancement of STY. Moreover, the application of a fed-batch dilution was reported to be better scalable and led to an increase of refolding titer by 34% for a two-chain immunotoxin (Linke et al. 2014). Fazeli et al. (2011) reported a refolding yield of 96% for IFNβ-1b when it was fed to the refolding tank with a final concentration of 10 µg/ml. Therefore, we propose that fed-batch refolding should be considered the method of choice. We are currently working on the development of a platform tool that generates process and mechanistic knowledge about the refolding procedure to finally correlate process data to product quality.
